# LUMINOUS database: lumbar multifidus muscle segmentation from ultrasound images

**DOI:** 10.1186/s12891-020-03679-3

**Published:** 2020-10-23

**Authors:** Clyde J. Belasso, Bahareh Behboodi, Habib Benali, Mathieu Boily, Hassan Rivaz, Maryse Fortin

**Affiliations:** 1 Department of Electrical and Computer Engineering, Concordia University, Montreal, H3G 1M8 Canada; 2 PERFORM Centre, Concordia University, Montreal, H4B 1R6 Canada; 3 Department of Diagnostic Radiology, McGill University, Montreal, H3G 1A4 Canada; 4 Department of Health, Kinesiology & Applied Physiology, Concordia University, Montreal, H4B 1R6 Canada; 5 Centre de recherche interdisciplinaire en réadaptation (CRIR), Constance Lethbridge Rehabilitation Centre, Montreal, H4B 1T3 Canada

**Keywords:** Ultrasound imaging, Paraspinal muscle, Lumbar multifidus muscle, Segmentation

## Abstract

**Background:**

Among the paraspinal muscles, the structure and function of the lumbar multifidus (LM) has become of great interest to researchers and clinicians involved in lower back pain and muscle rehabilitation. Ultrasound (US) imaging of the LM muscle is a useful clinical tool which can be used in the assessment of muscle morphology and function. US is widely used due to its portability, cost-effectiveness, and ease-of-use. In order to assess muscle function, quantitative information of the LM must be extracted from the US image by means of manual segmentation. However, manual segmentation requires a higher level of training and experience and is characterized by a level of difficulty and subjectivity associated with image interpretation. Thus, the development of automated segmentation methods is warranted and would strongly benefit clinicians and researchers. The aim of this study is to provide a database which will contribute to the development of automated segmentation algorithms of the LM.

**Construction and content:**

This database provides the US ground truth of the left and right LM muscles at the L5 level (in prone and standing positions) of 109 young athletic adults involved in Concordia University’s varsity teams. The LUMINOUS database contains the US images with their corresponding manually segmented binary masks, serving as the ground truth. The purpose of the database is to enable development and validation of deep learning algorithms used for automatic segmentation tasks related to the assessment of the LM cross-sectional area (CSA) and echo intensity (EI). The LUMINOUS database is publicly available at http://data.sonography.ai.

**Conclusion:**

The development of automated segmentation algorithms based on this database will promote the standardization of LM measurements and facilitate comparison among studies. Moreover, it can accelerate the clinical implementation of quantitative muscle assessment in clinical and research settings.

## Background

The paraspinal muscles (e.g. multifidus and erector spinae muscles) are a group of three muscles that originate from the occipital bone and continue down the spine to the sacrum [1]. Among the lumbar muscles, biomechanical studies have provided evidence for the importance of the lumbar multifidus muscle (LM) and its role in the dynamic stabilization and segmental control of the lumbar spine [2]. Over two thirds of the stiffness of the spine is attributed to the behaviour of the multifidi, establishing the LM’s importance in the neutral zone [3]. The neutral zone is described the range of intervertebral motion where spinal movement can occur with minimal internal resistance from the spine [4, 5]. As opposed to all the lumbar muscles, the LM has the characteristic of being a large multifascicular muscle that has a high cross-sectional area (CSA) [2, 4, 6]. As such, its structure allows for large forces to be generated over smaller ranges of operation [4]. This further supports the LM’s role of being a unit dedicated to providing lumbar spine stability [4, 7]. Therefore, the LM’s morphology (e.g. size, composition, asymmetry) and function (e.g. contractile ability) have become of great interest to researchers and clinicians involved in lower back pain (LBP) and muscle rehabilitation [2].

LBP is one of the most prevalent medical complaints, and it is estimated that between 60% to 80% of the population will experience at least one episode in their lifetime [8–10]. More importantly, the recurrence rate is extremely high and this common musculoskeletal condition is very disabling, and it severely affects the quality of life. Furthermore, it is projected to have an even higher personal and socio-economic burden as the world’s population ages [11, 12]. A large body of evidence confirmed that LM muscle structural changes (e.g. atrophy and increased in fatty infiltration) and functional deficits (e.g. decreased or increased contraction) occur in patients with LBP [13–16]. Along with LM and spinal dysfunction, such changes are also associated with lower physical function [17–20], poorer surgical outcomes [21, 22], and the recurrence of LBP symptoms [23, 24].

To date, magnetic resonance imaging (MRI), computed tomography (CT) scan, and ultrasound (US) have been used to quantify paraspinal muscle morphology. While MRI provides excellent soft tissue contrast and resolution and is the gold-standard imaging modality, it remains costly and its accessibility is limited. US is a portable, cost-effective, and non-ionizing imaging modality, providing a non-invasive method to obtain real-time in-vivo images for the assessment of LM morphology and function [25]. More specifically, US has been used to quantify the LM CSA, and CSA side-to-side asymmetry, as well as LM thickness in resting and contracted states to assess muscle activation (e.g. contraction) [26–28]. Additionally, measurements of the echo intensity (EI) can also be obtained using computer-aided gray scale analysis. EI has been investigated in studies related to muscle morphology, changes related to neuromuscular disorders, and studies investigating the relationship between muscle EI and size [29, 30]. Moreover, EI is used as an indicator of fatty infiltration and connective tissue which can be subsequently used to assess muscle quality [30–32].

Biomechanical modelling of the spine requires accurate measurements of the LM CSA for use in analytical processes that determine levels of LM wasting or injury [33]. In US, CSA measurements can be obtained by imaging the transverse section of LM [2]. The muscle’s border is then delineated from the rest of the surrounding tissue through manual segmentation. US examination requires training and experience, and the analysis and interpretation of the images are prone to subjectivity. Additionally, US assessments in the clinical setting are subject to issues concerning procedural and measurement reliability [25]. Procedural and measurement reliability are defined as the ability of an examiner to consistently and repeatedly perform the imaging procedure and measurements of the region of interest in the muscle, respectively [25]. However, due to the shape of the LM varying from one patient to another, and from one spinal level to another, examiners performing manual segmentations often encounter technical challenges affecting the quality and reliability of these measures. One of the major limitations of LM segmentation in US images is to determine the boundaries between the LM and the surrounding tissues [34]. Thus, the manual segmentation process of US images is highly rater-dependent, error prone, and can be labour intensive, which can limit its clinical applicability [35]. Therefore, the development of automated segmentation methods is warranted and would strongly benefit clinicians and researchers by decreasing the workload while simultaneously producing accurate and reliable segmentations that are comparable to expert manual segmentations [36].

The advent of deep learning has introduced many tools which are currently used to carry out various diagnostic tasks in medical US analysis. Moreover, as deep learning is being widely used in medical US analysis, its application continues to benefit from the ongoing research efforts made to further its state-of-the-art performance [36, 37]. Although recent efforts and studies have emphasized on US segmentation tasks using deep learning approaches, there is a limited amount of literature pertaining to the segmentation of skeletal muscle [37, 38]. Thus, it would be beneficial to support US segmentation tasks of musculoskeletal muscles such as the LM. Nevertheless, the development of automated segmentation methods requires manually annotated clinical datasets, which are currently scarce. Therefore, the purpose of this work is to provide a publicly available US database with the ground truth of the left and right LM at the L5 level, in both prone and standing positions, intended for the development of automated segmentation algorithms. To the best of our knowledge, this is the first publicly available US database of LM muscle.

## Construction and content

### Subjects’ description

The database contains 109 US datasets of young athletic adult volunteers who are involved in select varsity teams at Concordia University (64 males, 45 females, age: 21.1 ±1.7). The participants identified themselves among to the following choices for ethnical backgrounds: Black, White, Hispanic, and Other.

### Subjects’ characteristics

Subjects’ characteristics (sex, age [years], ethnicity, weight [kg], body mass index (BMI) [kg/m^2^], CSA [cm^2^], and mean EI) are listed in Table 1.

**Table 1:** Subjects’ characteristics and measurements of the right/left LM at the L5 level when subjects were either in the prone or standing positions

							Right MF Prone:			Left MF Prone:			Right MF Standing:			Left MF Standing:		
ID	Sex	Age	Ethnicity	Weight	Height	BMI	CSA	Mean	frame	CSA	Mean	frame	CSA	Mean	frame	CSA	Mean	frame
		[years]		[kg]	[m]	[kg/m^2^]	[cm^2^]	EI	#	[cm^2^]	EI	#	[cm^2^]	EI	#	[cm^2^]	EI	#
1	M	22	White	84	1.85	24.6	11.46	68.34	1	13.05	69.46	3	9.82	63.74	23	11.80	60.60	27
2	F	22	White	89	1.63	24.1	12.89	94.54	1	13.17	98.18	4	10.24	85.47	32	9.65	84.04	37
3	M	19	Black	73	1.78	23.0	6.95	33.06	1	7.25	33.68	1	8.75	40.19	22	7.98	23.24	22
4	F	21	White	88	1.62	27.3	8.89	63.14	1	8.72	59.69	1	9.21	64.11	29	9.31	54.35	29
5	M	23	White	86	1.78	28.2	8.68	86.29	1	8.19	84.63	1	7.40	65.77	28	8.80	66.18	28
6	F	22	White	95	1.86	25.6	7.79	67.50	1	6.96	59.28	5	9.98	57.78	28	9.81	46.34	32
7	F	23	White	64	1.62	24.1	7.46	44.32	2	6.40	44.74	4	10.01	54.16	24	10.14	57.95	26
8	M	21	White	87	1.77	27.5	10.68	48.21	1	9.68	44.67	5	12.34	27.43	29	ex	ex	ex
9	F	25	Black	76	1.80	23.5	10.56	70.40	1	9.94	82.77	3	10.57	53.37	26	11.27	61.31	31
10	M	21	White	88	1.87	27.1	9.89	52.15	1	11.17	54.94	4	11.50	37.47	33	10.70	48.93	34
11	M	20	White	82	1.88	21.9	8.26	79.01	1	7.48	72.55	3	ex	ex	ex	ex	ex	ex
12	F	23	White	89	1.73	23.4	10.23	72.46	1	11.69	74.34	4	ex	ex	ex	ex	ex	ex
13	M	20	White	81	1.85	24.9	13.17	64.68	1	13.43	58.56	5	ex	ex	ex	11.25	44.91	32
14	M	19	White	70	1.60	25.5	8.58	48.64	1	8.48	43.82	3	9.57	49.65	28	8.82	44.76	34
15	M	18	White	76	1.91	22.5	10.00	50.48	1	9.79	61.31	3	11.21	69.93	18	11.20	61.93	19
16	F	22	White	85	1.61	25.1	8.25	79.87	11	9.44	83.99	12	7.10	81.21	36	7.28	62.27	37
17	M	20	Black	83	1.77	26.6	9.21	31.17	1	9.25	43.04	5	11.29	39.09	33	10.40	34.36	37
18	M	22	White	87	1.74	24.8	8.50	50.98	1	8.77	53.05	5	9.09	53.48	23	9.61	52.55	31
19	F	23	White	76	1.65	24.6	10.35	85.04	1	10.30	82.09	4	ex	ex	ex	ex	ex	ex
20	F	21	White	71	1.94	26.3	8.49	77.54	1	7.37	72.83	1	9.64	63.46	22	9.25	52.45	22
21	M	21	White	87	1.68	26.9	9.25	47.25	1	10.16	52.29	6	10.81	49.18	27	10.82	46.62	32
22	F	22	White	99	1.69	25.0	6.66	70.99	5	7.18	77.96	5	8.91	84.42	22	8.59	79.93	22
23	M	20	White	81	1.72	27.3	12.66	41.62	1	11.79	39.47	4	10.40	51.32	29	10.83	44.37	30
24	M	21	Other	93	1.84	27.4	12.17	64.33	3	11.83	71.12	3	12.02	72.10	20	11.04	65.10	20
25	F	20	White	64	1.64	23.7	8.94	44.57	1	8.14	51.17	6	ex	ex	ex	9.14	39.66	39
26	M	19	White	95	1.80	29.3	9.74	44.58	1	10.02	38.41	4	ex	ex	ex	ex	ex	ex
27	F	19	White	67	1.62	25.6	6.50	40.24	1	6.73	47.87	1	9.06	50.93	24	7.71	42.21	24
28	M	22	White	90	1.90	25.0	13.10	52.43	3	13.47	53.25	5	13.91	53.12	30	ex	ex	ex
29	M	21	Asian	87	1.69	25.0	10.24	68.14	1	9.19	64.25	6	9.61	34.43	32	9.86	42.92	36
30	M	21	Black	85	1.78	27.0	15.50	40.57	1	14.06	32.05	3	15.67	27.29	25	15.93	30.84	26
31	M	22	White	85	1.89	25.1	10.90	55.20	1	12.08	48.72	4	11.94	33.51	27	11.55	36.26	30
32	F	21	White	86	1.63	22.8	9.94	79.33	1	9.22	70.38	6	11.72	65.04	31	10.95	61.74	32
33	F	22	White	86	1.64	28.1	10.36	63.54	1	9.32	73.73	1	9.91	66.21	22	8.95	54.39	22
34	F	23	White	85	1.68	22.0	7.86	67.51	1	7.06	56.19	3	8.74	50.89	24	9.14	43.63	28
35	F	19	Black	72	1.78	21.0	12.10	51.58	1	11.63	49.40	4	12.07	54.51	30	12.29	50.08	34
36	F	18	White	68	1.79	21.1	9.51	72.37	1	9.70	69.22	5	11.07	63.36	25	12.10	61.52	31
37	M	23	White	82	1.89	22.9	9.86	47.63	1	9.47	48.42	4	11.93	45.35	21	10.18	39.29	23
38	F	22	White	69	1.64	24.5	11.31	65.20	1	12.23	72.45	5	ex	ex	ex	ex	ex	ex
39	M	21	White	71	1.70	24.4	10.07	44.78	1	9.48	48.56	1	10.96	53.65	21	10.15	37.65	21
40	M	22	White	74	1.75	21.2	9.97	56.64	1	10.29	60.09	4	9.53	50.64	20	8.67	52.50	23
41	M	20	Black	80	1.72	27.2	10.63	29.76	2	9.21	37.47	4	10.24	35.99	28	9.33	36.53	29
42	M	19	White	79	1.86	22.9	11.02	63.70	6	10.39	66.68	3	12.64	62.74	24	13.71	58.63	28
43	M	19	White	65	1.76	28.1	12.68	55.90	1	11.82	61.36	5	ex	ex	ex	ex	ex	ex
44	F	18	White	71	1.73	27.1	9.03	74.50	1	8.30	79.54	5	ex	ex	ex	8.34	64.12	29
45	M	21	White	90	1.79	27.3	9.99	40.59	1	9.83	33.21	4	9.65	29.78	28	11.55	28.59	33
46	M	21	Black	79	1.75	26.0	10.29	24.27	1	10.24	24.69	1	12.44	25.97	28	ex	ex	ex
47	M	21	Other	85	1.72	28.8	7.89	65.95	1	8.87	63.56	1	10.11	83.03	18	9.15	82.92	18
48	M	24	White	61	1.78	26.9	10.15	58.76	1	9.88	54.96	3	11.03	46.84	29	10.93	47.86	33
49	M	19	White	89	1.86	21.9	10.67	44.40	1	8.11	24.36	4	9.05	37.54	22	10.63	22.68	24
50	M	18	White	80	1.86	20.1	10.47	31.63	1	10.06	39.22	3	10.98	31.76	17	11.35	29.59	18
51	M	22	White	71	1.79	21.7	11.10	57.40	1	10.29	52.81	3	13.32	71.22	26	13.08	57.04	27
52	M	23	White	70	1.70	24.4	11.18	56.90	1	9.83	46.86	4	11.00	34.40	30	10.61	27.71	32
53	M	20	White	65	1.83	18.1	ex	ex	ex	ex	ex	ex	12.04	69.94	29	ex	ex	ex
54	M	22	White	82	1.78	25.9	13.62	50.01	1	12.07	51.42	4	12.95	39.23	27	11.37	48.26	31
55	M	21	White	85	1.79	26.6	12.77	52.81	1	13.04	48.22	3	12.49	41.37	26	12.14	37.73	28
56	M	20	Black	84	1.82	25.5	9.63	61.60	1	9.68	70.77	5	10.18	44.26	30	9.76	28.30	31
57	F	22	White	64	1.70	27.4	9.65	75.99	1	8.69	69.11	6	ex	ex	ex	9.55	51.65	32
58	F	22	White	66	1.75	24.0	9.92	48.85	1	9.80	58.94	4	10.32	57.45	29	10.71	44.58	33
59	F	22	White	73	1.61	23.5	8.38	91.58	2	8.01	81.73	2	9.66	74.55	29	9.53	73.28	33
60	F	19	White	71	1.75	22.5	9.10	88.77	1	10.01	88.34	4	11.71	81.12	29	11.93	77.25	33
61	F	19	White	84	1.65	24.6	9.66	72.55	1	8.57	70.69	1	12.21	71.13	32	11.09	59.52	32
62	F	22	White	67	1.59	19.9	7.91	74.71	1	7.59	63.61	1	6.56	78.04	26	7.32	73.76	26
63	F	26	Black	93	1.82	28.2	9.78	53.99	4	10.82	62.93	5	11.71	61.23	25	11.84	53.36	29
64	F	19	White	63	1.65	19.7	9.96	61.81	2	9.29	63.05	4	11.67	53.61	24	11.44	49.30	29
65	F	18	White	67	1.79	24.6	9.48	42.82	1	10.29	52.33	4	11.38	49.93	26	ex	ex	ex
66	F	21	White	65	1.67	24.2	7.66	58.52	1	8.18	74.94	4	7.89	57.10	22	8.76	71.43	26
67	F	22	White	76	1.70	24.1	9.70	73.07	1	11.34	75.36	3	11.73	64.53	29	12.37	54.84	33
68	F	23	White	62	1.64	22.8	8.08	90.48	1	8.01	92.05	1	8.30	82.17	25	7.99	81.22	25
69	M	21	White	75	1.74	25.2	9.78	47.11	1	10.71	54.68	4	10.76	41.42	24	10.20	36.38	29
70	F	25	Black	88	1.80	27.3	8.84	60.70	7	8.26	47.65	9	9.71	48.71	27	9.16	48.22	29
71	M	21	White	95	1.77	30.2	10.44	54.50	1	10.73	47.97	4	11.79	38.85	24	12.34	29.49	24
72	M	22	White	87	1.76	28.2	10.96	53.85	1	12.01	60.47	5	10.96	41.79	33	12.11	39.97	34
73	F	22	White	67	1.52	22.4	6.02	56.33	1	5.57	64.45	1	ex	ex	ex	ex	ex	ex
74	F	19	White	67	1.66	20.3	ex	ex	ex	ex	ex	ex	6.25	62.77	25	7.48	61.64	29
75	M	25	White	79	1.79	27.7	9.40	36.00	1	9.84	40.28	3	9.89	35.10	29	11.13	49.59	32
76	F	22	White	61	1.83	26.4	11.48	54.43	1	11.18	55.93	1	11.65	56.13	28	12.16	52.79	30
77	M	18	White	79	1.76	22.8	7.16	37.07	1	6.65	50.92	4	ex	ex	ex	ex	ex	ex
78	M	22	White	79	1.67	28.4	10.98	57.33	1	11.27	62.22	1	11.05	48.14	22	11.57	36.71	22
79	M	21	Other	67	1.76	27.4	7.76	59.96	3	8.22	57.04	6	8.93	40.89	27	9.60	32.26	31
80	M	22	Other	74	1.81	22.5	11.09	44.77	1	10.99	40.52	3	ex	ex	ex	ex	ex	ex
81	M	20	White	100	1.99	25.3	10.51	68.27	1	9.50	60.57	5	10.49	59.12	37	9.31	47.17	37
82	M	20	White	69	1.87	25.1	11.19	67.54	1	11.71	67.55	5	12.13	49.37	37	13.08	39.16	38
83	M	23	Hispanic	71	1.73	23.7	11.69	40.85	1	10.62	41.66	3	ex	ex	ex	ex	ex	ex
84	M	21	White	82	1.78	26.0	9.64	69.55	1	10.12	60.27	3	9.58	68.19	21	10.78	65.43	24
85	M	21	Other	88	1.86	25.5	8.15	41.32	1	10.01	43.23	3	9.21	66.75	22	10.63	61.21	24
86	M	22	White	116	1.87	33.4	12.04	78.79	1	13.02	71.93	4	ex	ex	ex	ex	ex	ex
87	M	23	White	69	1.77	27.6	9.98	51.78	1	10.34	54.38	6	ex	ex	ex	ex	ex	31
88	M	22	Hispanic	82	1.81	24.9	7.02	38.89	1	6.50	34.42	3	8.19	35.91	31	8.47	15.79	31
89	M	21	White	54	1.74	24.2	9.62	52.12	1	8.94	49.39	4	12.35	46.17	19	10.83	43.71	21
90	M	19	White	50	1.75	24.4	11.79	49.62	1	10.51	60.19	3	11.25	46.32	27	12.00	57.42	28
91	M	20	White	92	1.85	27.1	13.36	52.17	1	13.67	52.08	3	14.12	54.75	27	13.49	46.13	29
92	F	18	White	56	1.67	21.2	8.33	70.57	1	6.96	75.23	4	ex	ex	ex	ex	ex	34
93	F	20	White	61	1.65	21.2	8.70	81.65	1	9.38	72.52	4	9.02	78.68	26	9.74	73.75	26
94	F	21	White	59	1.78	22.2	10.30	89.26	1	9.28	83.17	5	11.39	83.50	24	11.07	69.42	28
95	F	23	White	67	1.66	25.8	10.84	76.40	7	10.52	75.90	7	12.00	79.22	30	12.13	68.23	30
96	F	21	White	88	1.69	22.1	8.86	70.22	1	6.90	70.91	2	9.13	62.08	29	8.68	62.26	33
97	F	18	White	70	1.66	25.0	9.30	88.81	1	8.70	86.68	3	16.07	88.61	26	ex	ex	ex
98	M	21	White	58	1.89	23.7	11.08	39.14	1	ex	ex	ex	ex	ex	ex	ex	ex	ex
99	M	21	White	69	1.85	23.5	13.38	30.75	1	13.29	32.00	6	11.93	27.82	20	11.97	25.24	22
100	F	19	White	66	1.61	25.4	8.51	54.79	1	8.85	41.66	1	10.66	70.83	24	11.72	53.73	24
101	F	24	White	72	1.73	24.0	ex	ex	ex	12.54	79.20	6	11.29	53.02	29	10.15	61.42	35
102	M	20	White	102	1.82	30.9	10.69	77.85	1	10.73	74.16	1	10.71	88.79	26	8.69	81.02	26
103	F	23	White	86	1.64	24.0	7.47	92.36	1	8.85	89.39	1	ex	ex	ex	ex	ex	ex
104	M	23	White	87	1.82	26.1	ex	ex	ex	ex	ex	ex	10.85	50.18	23	11.60	54.79	26
105	M	22	White	73	1.79	22.9	9.96	63.73	1	9.99	56.18	3	ex	ex	ex	ex	ex	27
106	F	24	White	65	1.72	22.0	10.44	64.07	1	10.59	67.87	5	12.75	62.45	24	11.00	61.03	29
107	M	20	Black	96	1.76	31.1	10.58	23.84	1	9.36	30.74	1	10.86	25.85	23	11.92	38.00	23
108	M	21	White	84	1.85	24.6	9.92	59.90	1	10.98	62.37	4	9.89	66.62	27	11.08	62.32	28
109	M	22	White	65	1.78	25.8	9.32	41.32	1	9.94	37.75	4	9.56	24.38	29	10.55	33.94	33

ex = Excluded data

### US image acquisition

The 109 athletes underwent a US procedure to obtain LM images at the L5 level in both the prone and standing positions. The LOGIQ e ultrasound machine (GE Healthcare, Milwaukee, WI) was used with a curvilinear probe with its imaging parameters maintained at the following values for all image acquisitions: frequency: 5 MHz, gain: 60, depth: 8.0 cm [39]. Only the LM muscle was assessed, as it is the most commonly examined muscle amongst the paraspinal muscle group using US and is the most sensitive to spinal pathology. All data collection was performed by one of the investigators (M.F.) who applied a consistent and repeated technique throughout all image acquisitions: pressure was maintained on the adjacent hand and forearm handling the probe so as to prevent tissue deformation on the region of interest through transducer pressure. The acquisition of images in the prone position consisted in having the subjects lie in the prone position on a therapy table with a pillow underneath their abdomen to decrease lumbar lordosis [8]. To assess LM CSA, transverse US images were obtained bilaterally. For subjects with larger muscles, the right and left sides were imaged unilaterally. Similarly, LM CSA measurements were obtained in the standing position, where subjects stood in their habitual standing posture [39]. The images were stored as separate datasets for each subject in *.tif format.

### US image segmentation

The ground truth segmentations of LM CSA and LM EI measurements in prone and standing positions were performed on the acquired data using Fiji, a distribution of the ImageJ image processing software [40].

The ground truth segmentations for all measurements were manually obtained by one of the investigators (C.B.) who in preparation for this study, received training from another investigator (M.F.) with over 10 years of experience in spine imaging analysis. The inter-rater reliability between both investigators was examined on a set of 18 images and interclass correlation coefficient (ICC _2,1_) varied between 0.93-0.99. Images of subjects where the characteristic structures and landmarks of the LM could not be clearly distinguished were excluded from the database. All ground truth segmentations for each subject are available as binary masks and stored as separate *.tif files.

## Utility and discussion

### Database availability

The database is available at http://data.sonography.ai. The B-mode images and binary segmentation masks for each subject are deposited as *.tif files.

### Data organisation & file naming conventions

The database separates the B-mode images of each subject into a folder named “B-mode” and the masks into a folder named “Masks”. The datasets of subjects and corresponding binary segmentation masks are labelled with the same subject ID (1 to 109). The best available images (e.g. frames) for each subject were chosen for the segmentations. Since images were acquired bilaterally in some cases and unilaterally in subjects with larger muscles, different file naming conventions were used for the B-mode images as well as their corresponding masks. Table 1 can be used to verify whether a frame corresponds to either the right or left side, as well as whether the frame is in the prone or standing position.

#### Unilateral file naming conventions

**Fig. 1:**
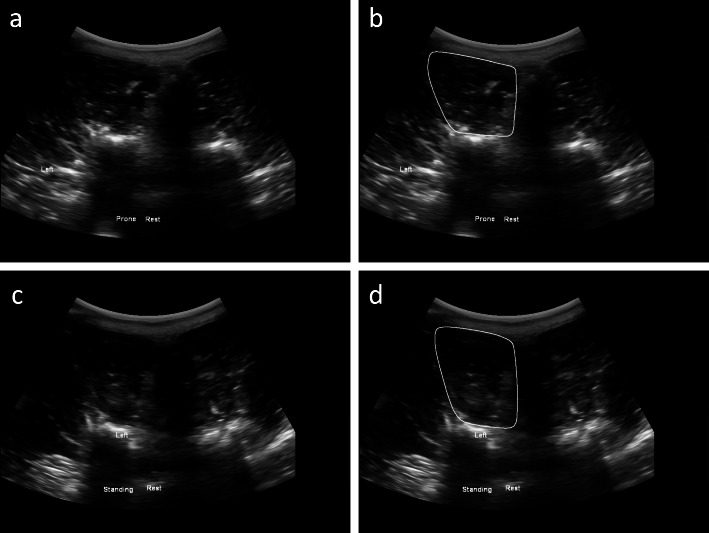
(**a**) B-mode image of subject 50 (acquired unilaterally) with corresponding segmentation of the left MF in the prone position shown in (**b**). (**c**) B-mode image of subject 50 (acquired unilaterally) with corresponding segmentation of the left MF in the standing position shown in (**d**)

For the subjects where the images were acquired unilaterally, the B-mode images and masks have a one-to-one correspondence. The file names for the B-mode images and masks have the following generic format: *X_Y_Bmode.tif* and *X_Y_Mask.tif*, where *X* is the subject ID, and *Y* is the frame number. As an example, *50_3_Bmode.tif* would have a corresponding mask *50_3_Mask.tif*. This can be seen in Fig. 1a and b.

#### Bilateral file naming conventions

**Fig. 2:**
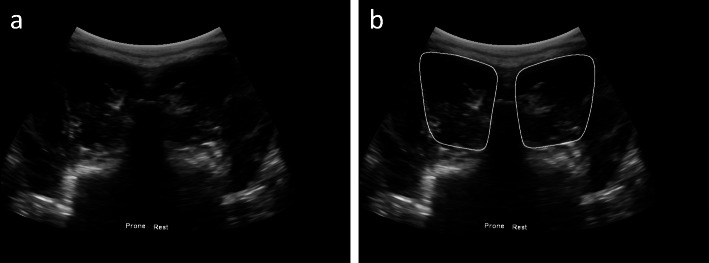
(**a**) B-mode image of subject 46 (acquired bilaterally) with corresponding segmentations of the left and right MF in the prone position shown in (**b**)

For the subjects where images were acquired bilaterally, the file names for the B-mode images and masks have the following generic format: *X_Y_Bmode.tif* and *X_Y_MaskZ.tif*, where *X* is the subject ID, *Y* is the frame number, and *Z* is a value of 1 or 2 used as an identifier to distinguish between the right and left side, respectively. As an example, *46_1_Bmode.tif* would have corresponding masks *46_1_Mask1.tif* and *46_1_Mask2.tif*. This can be seen in Fig. 2a and b.

### Discussion

Due to portability, cost-effectiveness, and efficiency, clinicians and researchers widely use US as an imaging modality in their screening and diagnostic procedures over other imaging modalities such as MRI, CT and X-ray. However, US presents its own set of disadvantages relating to the task of manual segmentation. Due to speckle noise in US images, manual segmentation is highly rater-dependent and thus, is susceptible to errors which affect LM analysis and results. As such, the development of powerful segmentation algorithms can help mitigate the aforementioned issues. Deep learning techniques can be employed to extract features from the data and can then be used to perform automatic US image segmentation [41]. Although potential applications of deep learning algorithms have been demonstrated for MRI and microscopy modalities, very few have focused on algorithms applied to US [37]. Furthermore, the performance of deep learning algorithms is highly dependent on a high volume of quality data. The availability of public repositories on clinical data pertaining to LM muscle images are scarce, and thus greatly limit the development and testing of the segmentation algorithms. As such, our aim with this study was to provide the first publicly available US database of the LM.

This database is comprised of 109 subjects with the ground truth of the left and right LM at the L5 level in both prone and standing positions. The ground truth data can enable the development of deep learning algorithms used for automatic segmentation tasks related to the LM. Given the volume of the annotated data, the developed algorithm can have a better generalization capability through proper parameter tuning and data augmentation [41]. Moreover, deep learning algorithms can exploit the morphological features that trained experts use to perform their segmentations [41, 42]. Furthermore, the algorithms can produce comparable results to those of the examiner [36, 43]. As such, examiner subjectivity during assessment of muscle morphology can be reduced. In addition, it would greatly benefit clinicians and researchers whilst enabling them to perform assessments in a practical and time-efficient manner.

This database contributes and dedicates itself to advancing the development of automatic segmentation algorithms related to the assessment of LM muscle morphology. However, this database only includes young athletic adults aged between 18 and 26 years old. Within the dataset, there are natural variances in age, BMI, and other underlying conditions which may differ from one participant to the next. As such, algorithms which are developed using our dataset should be mindful of these limitations and foresee difficulties in accurate segmentation when subjected to samples from other populations, a problem commonly referred to as domain shift. Thus, future efforts need to be made to extend this database to include sedentary and older adults, which are more representative of the general population suffering from LBP. When viewing US image of younger muscle (e.g. higher fluid content), contrasting echogenicities of hypoechoic (toward black) muscle and hyperechoic (toward white) fascia allow for easier tissue differentiation and identification of key landmarks [44]. With ageing, there is a natural increase in fibrous tissue and thus the distinction between muscle and fascia is more difficult [25, 45]. As such, this database should be treated as a platform of an ongoing process towards the automatization and standardization of LM muscle measurements from US images.

## Conclusion

Herein, we presented the LUMINOUS database which contains manual segmentations of LM images at the L5 level obtained via US as well as their corresponding binary masks. The database is comprised of 109 datasets, which will enable the development of automated segmentation algorithms of the LM. This database will provide a means to support the standardization of US measurements, facilitate comparison between studies and accelerate the clinical implementation of quantitative muscle assessment in clinical and research settings.

## Data Availability

The database presented in this study is freely available online at http://data.sonography.ai.

## Abbreviations

US: Ultrasound
LM: Lumbar multifidus
CSA: Cross-sectional area
LBP: Lower back pain
MRI: Magnetic resonance imaging
CT: Computed tomography
EI: Echo intensity
BMI: Body mass index
